# Asthma control and sputum eosinophils in adult patients: a cross-sectional study in southern Brazil

**DOI:** 10.1038/s41598-023-48381-1

**Published:** 2023-12-05

**Authors:** Vanessa Albano Barcellos, Vanessa Cristina Hartmann dos Santos, Maria Ângela Fontoura Moreira, Paulo de Tarso Roth Dalcin

**Affiliations:** 1 Programa de Pós-Graduação em Ciências Pneumológicas, Faculdade de Medicina, Universidade Federal do Rio Grande do Sul (UFRGS), Ramiro Barcelos, 2400, Porto Alegre, RS 90035-003 Brazil; 2 Serviço de Pneumologia, Hospital de Clínicas de Porto Alegre, Ramiro Barcelos, 2350, Porto Alegre, Brazil

**Keywords:** Cytological techniques, Biomarkers

## Abstract

Asthma control and health related quality of life are an important goal of asthma management, but their association with sputum eosinophilic inflammation has been less firmly established. To investigate the relationship of asthma control and quality of life with sputum eosinophils in clinical practice. Cross-sectional study with a convenience sample, including patients with asthma, aged between 18 and 65 years, attending to outpatient clinic. Patients underwent sputum induction, pulmonary function tests, Juniper’s Asthma Quality of Life Questionnaire (AQLQ), Asthma Control Test (ACT), Global Initiative for Asthma (GINA) criteria for evaluation of asthma control and severity of the disease, blood count analysis, serum IgE and cutaneous prick test. Sputum sample was considered as eosinophilic if the percentage of eosinophils was ≥ 3%. A total of 45 individuals were enrolled, 15 with eosinophilic sputum (≥ 3% eosinophil cells) and 30 with non-eosinophilic sputum (< 3% eosinophil cells). There were no association of ACT an AQLQ scores with sputum eosinophilia (p > 0.05). This study suggested that the finding of sputum eosinophilia was not related to asthma control neither with health-related quality of life in patients with severe asthma.

## Introduction

Asthma is a common, chronic respiratory disease affecting 1–18% of the population in different countries, characterized by variable symptoms of wheeze, shortness of breath, chest tightness, cough, and by variable expiratory airflow limitation. It is a heterogeneous disease which consists in multiple phenotypes that are distinguished by clinical, function and inflammatory characteristics. The eosinophil is one of the most important cells related to airway inflammation in asthma^1^.

The type 2 asthma phenotype is based on an eosinophilic inflammation that can occur in the absence of an allergic reaction. This involves biomarkers such as Interleukin (IL) 4, IL-5 and IL-13, Immunoglobulin E (IgE), fractional exhaled nitric oxide (FeNO), peripheral blood eosinophils and eosinophils in induced sputum^2^. These biomarkers can be employed in several ways to aid treatment decisions^3^.

Treatment of asthma can reduce or remove symptoms. The assessment of asthma control (control of symptoms and risk of adverse outcomes) is determined by the interaction between the patient’s genetic background, environment exposure, psychological factors, treatments, and the underlying disease process^1^.

The sputum induction method has been validated and standardized, providing a safe and relatively noninvasive way to collect material from the lower airways^4,5^. On the other hand, the method of analyzing the samples is laborious and requires well-trained technicians using highly specialized laboratory equipment^6^.

The importance of examination of sputum cellularity in the management of moderate-to-severe persistent asthma has grown as recent studies have demonstrated that the number of severe exacerbations is lower when induced sputum findings are used to design the anti-inflammatory treatment than when the treatment is based on the current guidelines^7^.

Considering that asthma control is an important goal of asthma management and that its association with sputum eosinophilic inflammation has been less firmly established, in this exploratory study we sought to analyze the implementation in our institution of conventional method for sputum processing in adult patients with severe asthma, investigating the relationship of asthma control and quality of life with sputum eosinophils in clinical practice.

## Methods

### Study design

This was a cross-sectional study approved by the ethics and research committee of the Hospital de Clínicas de Porto Alegre (HCPA), protocol number 5.860.232, and Plataforma Brazil, protocol number 5.860.232. All patients signed an informed consent form. Part of the results has been published previously as an original article^8^. The population studied in the present study was a convenience sample, with no sample size calculation.

Secondarily, this was an exploratory study in which we sought to analyze the preliminary implementation in our institution of conventional method for sputum processing in adult patients with severe asthma. Exploratory research design is conducted for a research problem when the researcher has no past data or only a few studies for reference^9^.

### Population

Patients were recruited from the Asthma Outpatient Clinic of HCPA, Porto Alegre, Rio Grande do Sul, Brazil. This Clinic is responsible of the care of patients with severe asthma.

The study included patients aged between 18 and 65 years. The diagnosis of asthma was confirmed by compatible history and evidence of reversible airflow obstruction in spirometry: forced expiratory volume in the first second (FEV_1_) less than 80% of the predicted value and FEV_1_/forced vital capacity (FVC) ratio less than 75% plus substantial improvement in airflow after inhalation of short-acting beta_2_-agonist bronchodilator (BD) (increase in FEV_1_ greater than 12% in relation to the pre-BD value and greater than 200 mL in absolute value or increase in FEV_1_ greater than 20% and exceeding 250 mL spontaneously over time or after intervention with medication)^1^.

Asthmatic subjects exposed to smoking were included. Individuals who reported smoking in the last 30 days and had a smoking index greater than 5 pack-years were considered active smokers. Smokers in cessation or ex-smokers were defined as individuals who had quit smoking in the last 30 days or more and had a smoking index greater than 5 pack-years. Nonsmokers were defined as individuals who reported never having smoked and ex-smokers with a smoking index of less than 5 pack-years. The smoking index (pack-years) was calculated as follows: number of cigarettes smoked per day/20 × the number of years the person had smoked.

The exclusion criteria from the study were: pregnant women; patients with other chronic lung diseases such as bronchiectasis, sequelae of pulmonary tuberculosis, diffuse lung fibrosis; lung neoplasm or neoplasm of other sites; human immunodeficiency syndrome, acquired immunodeficiency syndrome, or congenital immunodeficiency syndrome; psychiatric illness or incapacitating chronic neurological disease that could prevent the performance of the study procedures; and patients who did not complete the study evaluation tests.

### Procedures

Induced sputum samples were collected to evaluate cellularity, following institutional protocols. We considered the sputum sample as eosinophilic if the percentage of eosinophils was ≥ 3%^10^.

The level of asthma control was assessed using the Asthma Control Test (ACT)^11^ and the GINA table^1^.

Spirometry was performed using a Jaeger v 4.31a spirometer (Jaeger, Wuerzburg, Germany). The carbon monoxide diffusing capacity (DL_CO_) was measured by a single sustained breath of a special gaseous mixture, using Master Screen Diffusion equipment (Jaeger, Wuerzburg, Germany). Pulmonary volumes were measured using the Master Screen Body-Plets (Jaeger, Wuerzburg, Germany)^12,13^.

All the patients were asked about Health-related Quality of Life using Juniper’s Asthma Quality of Life Questionnaire* (*AQLQ)^14,15^. The AQLQ comprises four domains: symptoms, activity limitation, emotional function, and environmental exposure. Each domain is scored from 1 to 7; scores of 1 indicate maximal impairment and scores of 7 indicate no impairment.

Blood count analysis was performed, total eosinophils count, and percentage was used. We consider blood count > 300 cell/mm^3^ as eosinophilia^16^. Serum IgE dosage was analyzed and considered high if greater than 100 IU/mL, according to the reference value^17^. The skin prick test for allergy was conducted according to published protocols, if the patient had at least one cross positive, it was considered positive test^18^.

Patients were asked about symptoms of allergic rhinitis and were classified about its control.

### Statistical analysis

This study was made with a convenience sampling. All data were processed and analyzed using the Statistical Package for the Social Sciences, version 22.0 (SPSS Inc., Chicago, IL, USA). Quantitative variables were expressed using mean and standard deviation or median and interquartile range. Categorical variables were expressed using absolute and relative frequencies.

The associations between categorical variables were analyzed using Pearson’s chi-square test or Fisher’s exact test. To compare means, we used Student’s t-test or one-way analysis of variance, supplemented by Tukey’s test. In case of asymmetry, Mann–Whitney or Kruskal–Wallis tests were used and supplemented by Dunn’s test. A p-value ≤ 0.05 was considered statistically significant and all tests were two-tailed.

### Ethics statement

This was an exploratory study using data of a cross-sectional study, approved by the ethics and research committee of the Hospital de Clínicas de Porto Alegre (HCPA) and Plataforma Brasil (protocol number 1.139.117). All patients signed an informed consent form. The authors signed a data use agreement protecting the confidentiality of patient information. All study methods were carried out in accordance with international and national guidelines and regulations for clinical studies of humans (Declaration of Helsinki and Brazilian Governmental regulation—Plataforma Brasil).

## Results

**Figure 1:**
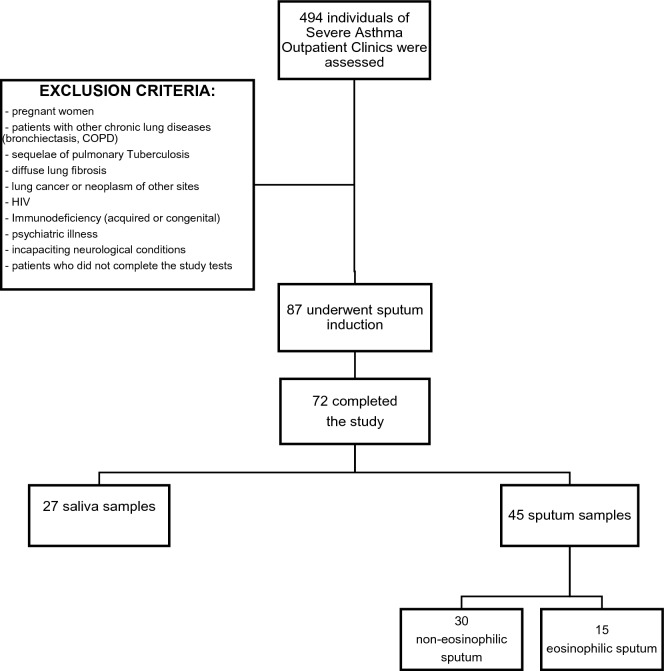
Flow diagram of study selection and exclusion criteria.

During a period of 20 months, a total of 494 adults subjects were assessed in the initial evaluation. Out of them, 87 fulfilled all the criteria, but 15 withdraw consent to sputum induction. Thus 72 subjects completed the study (see Fig. 1—Flow diagram of study selection and exclusion criteria).

Despite performing the protocol correctly, 27 individuals could not achieve the necessary volume of sputum to do the analyses. We classified them as non-adequate sputum sample. Table 1 shows the demographic, clinical data, and comparison of patients in groups with inadequate sputum sample and with adequate sputum sample. The individuals with inadequate sputum sample were younger than those with adequate sputum sample (respectively, 42.1 ± 12.9 vs 49.1 ± 12.5 years, p = 0.027). There was no other statistically difference between the 2 groups.

**Table 1:** Characteristics of study sample, comparing groups between those who got saliva and sputum.

Variable	Non-adequate sample (saliva) (N = 27)	Adequate sputum sample (N = 45)	P-value
Sex, female n (%)	22 (81.5)	35 (77.8)	0.713
Age (years), mean ± SD	42 ± 2.5	49 ± 1.9	0.027
Age of asthma onset (years), median (IR)	4 (0–16)	5 (0–35)	0.046
BMI (kg/m^2^), mean ± SD	30.7 ± 1.3	31.1 ± 1	0.76
Ethnicity, white n (%)	23 (85.2)	33 (73.3)	0.224
Tobacco exposure, n (%)			0.1
Never smoked	21 (77.8)	27 (60)	
Current smoker	2 (7.4)	5 (11.1)	
Former smoker	4 (14.8)	13 (28.9)	
Charlson’s index (points), median (IR)	1 (0–2)	2 (0–4)	0.136
ACT (points), median (IR)	15 (6–24)	16 (8–24)	0.576
ACT (≥ 20 points; controlled), n (%)	6 (22.2)		
Lung function, mean ± SD			
FVC (L)	2.9 (0.1)	2.7 (0.1)	0.316
FVC%	83.8 (3.4)	79.2 (2.7)	0.299
FEV1 (L)	2 (0.1)	1.8 (0.1)	0.126
FEV1%	67.9 (3.4)	63.5 (2.8)	0.324
FEV1/FVC%	64.4 (3.2)	64.9 (1.6)	0.884
Severity of asthma, n (%)			0.099
Mild	1 (3.7)	0	
Moderate	3 (11.1)	1 (2.2)	
Severe	23 (85.2)	44 (97.8)	
Serum eosinophils, mean ± SD	2.9 ± 0.4	3.4 ± 0.4	0.445
Serum eosinophilia (> 300), n (%)	11 (40.7)	15 (33.3)	0.533

*SD* standard deviation, *IQR* inter-quartile range. *BMI* body mass index, *FVC* forced vital capacity, *FEV1* forced expired volume at first second. Lung function is expressed by pre-Bronchodilator spirometry. Severity of Asthma was classified according to GINA.

A total of 45 individuals were enrolled in the study (group with adequate sputum samples). Thirty-five patients (77.8%) were female, the mean age was 49.1 ± 12.5 years, the mean BMI was 31.2 ± 6.8 kg/m^2^ and 73.3% were white. The median ACT was 16 (13–21) points. The mean FVC was 79.5 ± 15.8% of predict, the mean FEV_1_ was 66.3 ± 18.0% of predict and the mean FEV_1_/FVC was 67.9 ± 11.0%. Ninety-eight percent of subjects were classified as severe asthma (steps 4 and 5 of GINA classification).

Those patients with adequate sputum samples were divided in two groups: 15 individuals with eosinophilic sputum (≥ 3% eosinophil cells) and 30 with non-eosinophilic sputum (< 3% eosinophil cells). The comparison of demographic and clinical data between patients with non-eosinophilic and eosinophilic sputum is showed in Table 2. There were no statistically significant differences between groups. In both groups female sex was predominant, and the main ethnicity was white. When asthma control was assessed according to GINA, only 13% of all patients had well controlled asthma. The median of ACT score was 15.5 (13–20) in the non-eosinophilic and 16 (12–23) in the eosinophilic sputum group, p = 0.621. The ACT score was ≥ 20 points in only 9 (20%) of patients in non-eosinophilic sputum group and in 6 (13.3%) of patients in eosinophilic sputum group, p = 0.513.

**Table 2:** Characteristics of the groups and comparison between non-eosinophilic and eosinophilic sputum.

Variable	Total (N = 45)	Sputum eosinophils < 3% (N = 30)	Sputum eosinophils ≥ 3% (N = 15)	P-value
Sex, female N (%)	35 (77.8%)	25	10	0.258
Age (years), mean ± SD	49 ± 1.9	49.3 ± 1.8	48.7 ± 4.8	0.91
Age of asthma onset (years), median (IR)	5 (0–35)	5.5 (0–36.5)	5 (0–33)	0.903
Ethnicity, white N (%)	33 (73.3%)	22	11	1
BMI (kg/m^2^), mean ± SD	31.7 ± 1	30.9 ± 1.4	31 ± 1.5	0.851
Tobacco exposure, n (%)				0.645
Never smoked	27 (60%)	17	10	
Smoker	5 (11%)	4	1	
Ex-smoker	13 (28.9%)	9	4	
Severity of asthma, N (%)				0.486
Moderate	1 (2.2%)	1	0	
Severe	44 (97.8%)	29	15	
Allergic rhinitis, N (%)				0.066
No	N/A	N/A	N/A	
Controlled	36 (80%)	22	14	
Not controlled	9 (20%)	8	1	
Asthma control, N (%)*				0.492
Well controlled	6 (13.3%)	3	3	
Partially controlled	16 (35.6%)	10	6	
Uncontrolled	23 (51.1%)	17	6	
ACT (points), median (IR)	16 (8–24)	15.5 (8.5–22.5)	16 (8–24)	0.621
ACT (≥ 20 points; controlled), n (%)	15 (33.3%)	9	6	0.513

*SD* standard deviation, *IR* interquartile range, *BMI* body mass index.

*According to GINA.

**Figure 2:**
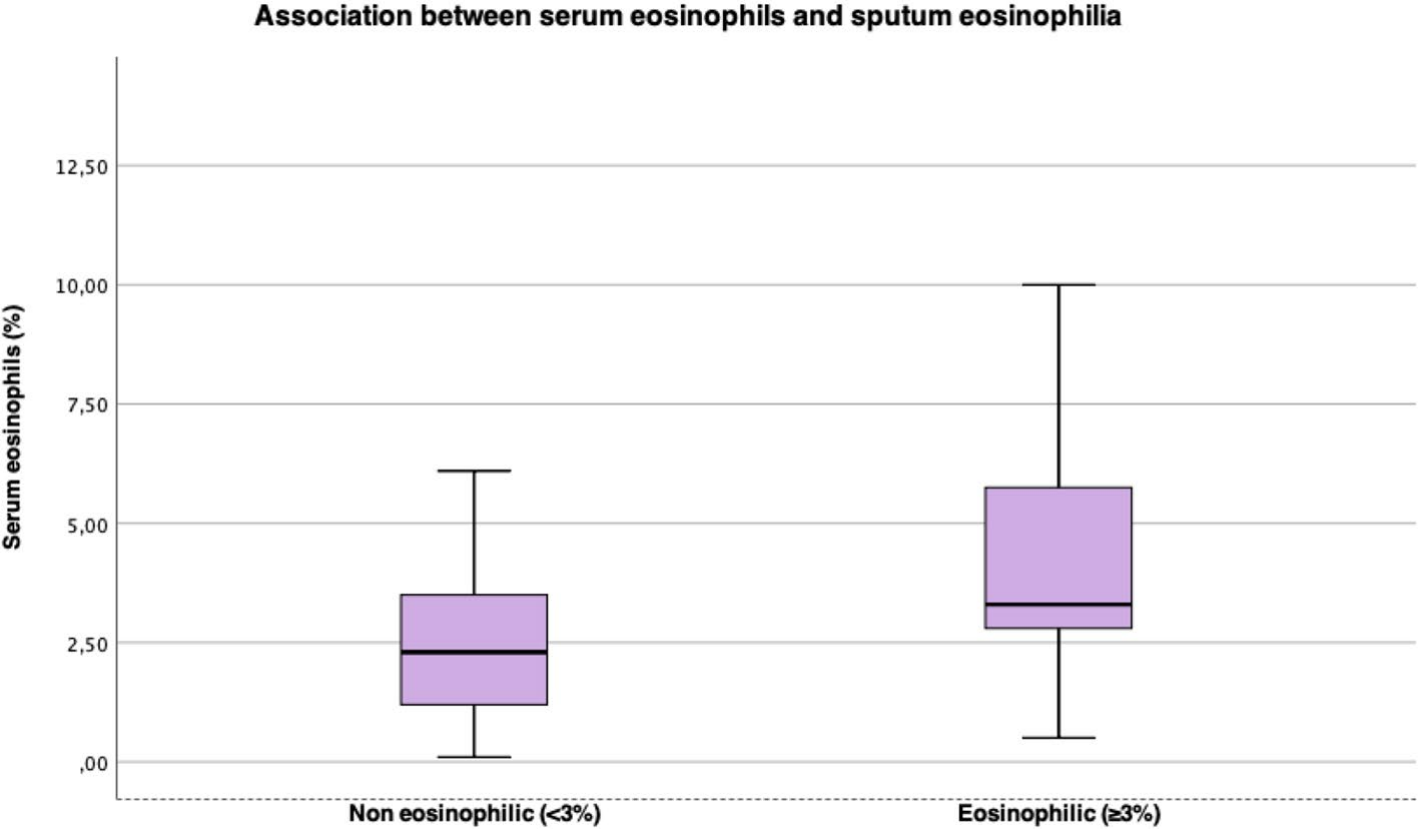
Association between serum eosinophils and sputum eosinophilia.

Table 3 presents comparison between lung function, cutaneous prick test, serum eosinophils and serum IgE between patients with non-eosinophilic and eosinophilic sputum. The individuals with eosinophilic sputum had higher serum eosinophils (%) levels than the non-eosinophilic group (respectively, 3.3% vs 2.1%, p = 0.037) and when considering serum eosinophilia as eosinophils count > 300 mm^3^, there was association with sputum eosinophilia too (p = 0.044), Fig. 2. There were no other significant statistical differences between groups. The proportion on patients with blood eosinophilia was 33.3%.

**Table 3:** Comparison between lung function, cutaneous prick test, serum eosinophils and serum IgE between patients with non-eosinophilic and eosinophilic sputum.

Variable	Total (N = 45)	Sputum eosinophils < 3% (N = 30)	Sputum eosinophils ≥ 3% (N = 15)	P-value
Lung function, mean ± SD				
FVC (L)	2.8 ± 0.1	2.6 ± 0.1	3.1 ± 0.3	0.198
FVC (% predicted)	80.2 ± 2.5	77 ± 2.8	85.7 ± 4.1	0.067
FEV1 (L)	1.9 ± 0.1	1.8 ± 0.1	2.1 ± 0.2	0.441
FEV1 (% pred.)	66.5 ± 2.8	64.8 ± 3.6	69.5 ± 4.3	0.366
FEV1/FVC %	67.3 ± 1.7	68.1 ± 2.2	66.1 ± 2.8	0.353
DLCOcSB (mmol/min/kPa)	6.3 ± 0.3	6.1 ± 0.4	6.6 ± 0.4	0.308
DLCOcSB (%)	74.7 ± 3,1	74.6 ± 2.5	78.4 ± 4.8	0.302
TLC (L)	5.9 ± 0.2	5.8 ± 0.2	6 ± 0.4	0.838
TLC (% pred.)	113 ± 2.2	113.8 ± 2.8	111.7 ± 3.8	0.981
FRC (L)	5.9 ± 0.2	3.7 ± 0.9	3.6 ± 0.3	0.05
FRC (% pred.)	113.1 ± 0.2	135.4 ± 6,1	124.8 ± 7.6	0.815
RV (L)	3 ± 0.2	3.1 ± 0.2	2.9 ± 0.3	0.791
RV (% pred.)	171.1 ± 6.8	174.6 ± 8.6	174.6 ± 8.7	0.8
Cutaneous prick test, n (%)				
At least one positive test	23 (51%)	13	10	0.208
*D. pternonyssinus*	20 (44.4%)	12	8	0.527
*Blomia tropicalis*	17 (37.8%)	9	8	0.193
Domestic dust	17 (37.8%)	10	7	0.517
Histamine	45 (100%)	30	15	
Serum eosinophilia (> 300 eos), n (%)	15 (33.3%)	7	8	0.047
Other, median (IR)				
Serum eosinophils (× 10^3^/mL)	0.23 (0–0.4)	0.2 (0–0.2)	0.3 (0–0.7)	0.077
Serum eosinophils (%)	3 (0.3–5.8)	2.1 (0–2.1)	3.3 (0–7.6)	0.037
Serum IgE (UI/mL)	113 (0–492.5)	109 (0–423.1)	106 (0–553)	0.931

*SD* standard deviation, *IR* interquartile range, *FVC* forced vital capacity, *FEV1* forced expired volume at the first second, *DLCOcSB* carbon monoxide diffusion, *TLC* total lung capacity, *FRC* functional residual capacity, *RV* residual.

**Figure 3:**
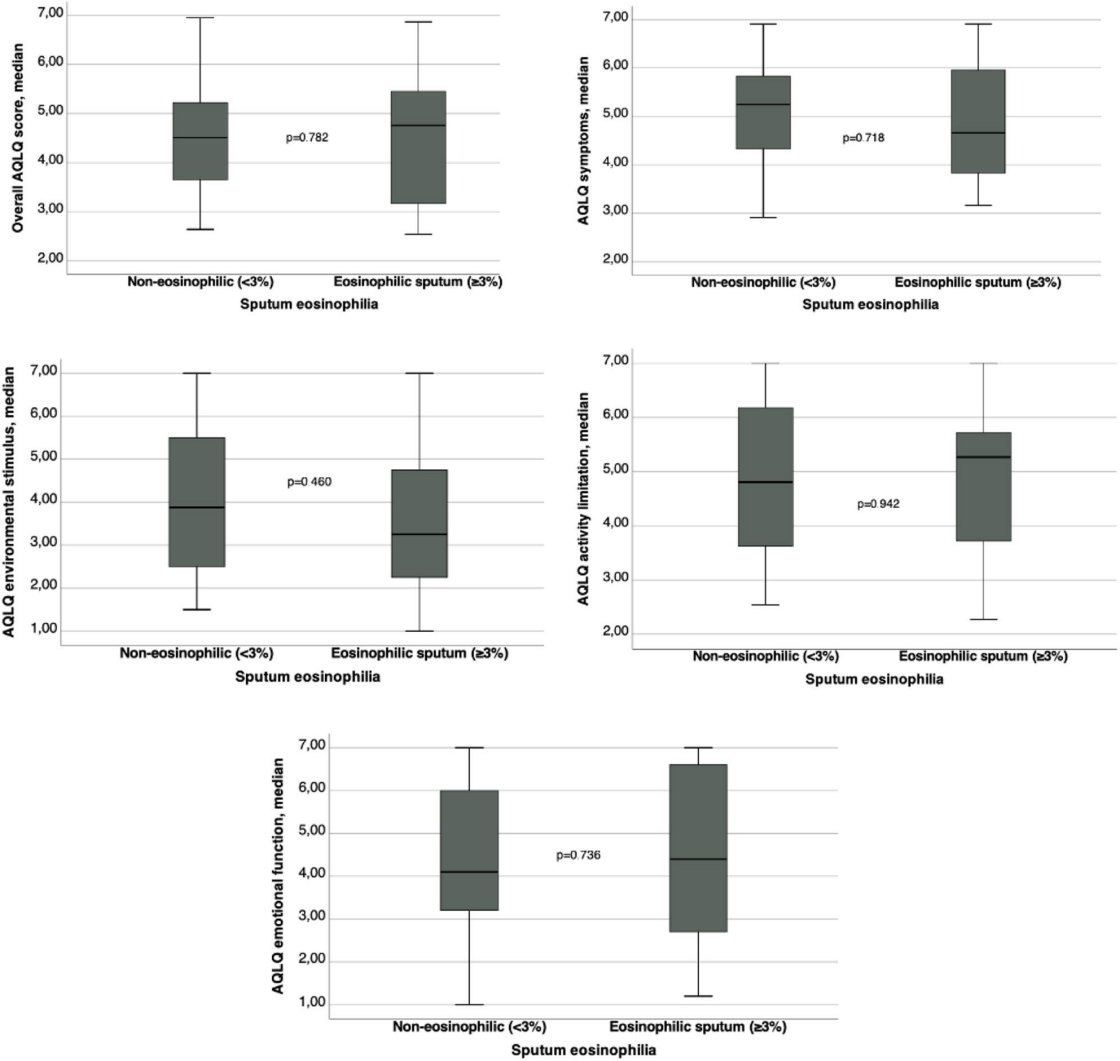
Comparison between AQLQ domain scores.

Table 4 shows comparison between ER visits, hospital admissions and AQLQ domain scores between patients with non-eosinophilic and eosinophilic sputum. Graphics comparing AQLQ scores are on Fig. 3. There were no significant statistical differences between groups for ER visits, estimated number of days away from job or school, number of hospital admissions and scores of AQLQ. The ER visits and hospital admission rates were low in both groups in the present study. The AQLQ domain scores showed a moderate impairment in quality of life in both groups.

**Table 4:** Comparison between emergency room visits, hospital admissions and AQLQ domain scores between patients with non-eosinophilic and eosinophilic sputum.

Variable	Sputum eosinophils < 3% (N = 30)	Sputum eosinophils ≥ 3% (N = 15)	P-value
Visited the ER because of asthma in the last 4 weeks (yes), n (%)	1 (2.2)	2 (4.4)	0.254
Visited the ER in the last year (yes), n (%)	15 (33.3)	6 (13.3)	0.751
Number of visits to the ER because of asthma in the last 4 weeks, median (IR)	0 (0–0)	0 (0–0)	0.967
Number of visits to the ER because asthma in the last year, median (IR)	1 (0–3.25)	0 (0–2)	0.312
Estimate number of days away from job or school in the last year, median (IR)	0 (0–8.5)	0 (0–2)	0.756
Number of hospital admissions in the last year, mean ± SD	0.4 ± 0.2	0	0.526
Overall AQLQ score, median (IR)	4.5 (3.6–5.3)	4.8 (3.1–5.5)	0.782
AQLQ symptoms, median (IR)	5.3 (4.3–5.8)	4.7 (3.8–6.0)	0.718
AQLQ activity limitation, median (IR)	4.8 (3.6–6.2)	5.3 (3.2–5.7)	0.942
AQLQ emotional function, median (IR)	4.1 (3.2–6.0)	4.4 (2.2–6.6)	0.736
AQLQ environmental stimulus, median (IR)	3.9 (2.5–5.5)	3.3 (2.0–5.3)	0.460

*ER* emergence room, *AQLQ* asthma quality of life questionnaire.

## Discussion

The knowledge about sputum cellularity in inflammatory respiratory diseases allows more comprehensive approach and care for patients with moderate or severe conditions^7^. This cross-sectional study suggested that the finding of eosinophilic sputum was not related to asthma control neither with health-related quality of life in a population with severe asthma attending to an asthma outpatient clinic in a tertiary and academic hospital in Southern Brazil.

Duncan et al. used the measures of inflammatory cells in induced sputum to search for asthma severity relationship with eosinophils. This was research with chronic stable asthma^19^ which included former smokers, comparing forced expiratory volume, symptoms, and sputum eosinophils apoptosis. It was demonstrated that reduced eosinophil apoptosis and sputum eosinophil load are correlated with degree of self-reported symptoms and severity of the disease.

In a retrospective longitudinal study of 187 patients, Demarche et al. demonstrated that asthma control was associated with fluctuations in sputum eosinophilic inflammation^20^. Furthermore, they have calculated a minimal important increase and decrease in sputum eosinophils associated with a change of at least 0.5 in the Asthma Control Questionnaire (ACQ). The age and smoking status were like the present study, but the asthma severity was lower than that identified in our study.

Pizzichini et al. studied a population of 130 patients with asthma that had been receiving treatment for asthma for at least a year and were lifelong non-smokers or ex-smokers, who were assessed to induced sputum and to answer quality of life questionnaires^21^. The sputum was labelled eosinophilic if sputum eosinophils were ≥ 3%, neutrophilic if neutrophils were ≥ 60%, and pauci-granulocytic if neither. It was found that near 70% of subjects with controlled asthma had pauci-granulocytic sputum, suggesting that answering “No” to the four questions of GINA table (1) is a good indicator of control of air inflammation. On the other hand, in patients with partially controlled or uncontrolled asthma, there was no difference between the groups with eosinophilic, neutrophilic, or pauci-granulocytic sputum. The population of this research differs from ours in some features: they did not include active smokers and they had number of individuals with controlled asthma higher than ours. In our population, with GINA criteria, only 6.7% had control of the disease.

Recently, Athanazio et al. evaluated prevalence of eosinophilic phenotype in patients with severe asthma, with blood eosinophils count. The prevalence of patients with severe asthma and eosinophils > 300 cell/mm^3^ in Brazil was 40%^16^. Our research found similar data, 33% of our severe asthma population studied had sputum eosinophilia (defined as sputum eosinophils ≥ 3%). When comparing serum and sputum eosinophils, blood eosinophils count > 300 cell/mm^3^ was associated with sputum eosinophilia (p = 0.044), secondarily there was association between percentual of serum eosinophils and sputum eosinophilia.

Our study has some limitations. First, this was a cross-sectional study, so it was not possible to establish a temporal link between sputum eosinophilia and asthma control and health related quality of life. Second, the sample size was too small with a convenience sample and would be associated with low statistical power. Third, our study did not encompass all the spectrum of asthma severity, and the study only included steps 4 and 5 of GNA severity classification. Also, the study population was selected from patients referred to a reference center and was probably biased toward the more severe disease. It is important to emphasize that the patients in our sample were on high doses of inhaled steroids, the majority was using more than 1600mcg of budesonide. Fourth, the study population includes only those patients who were able to produce a sputum sample of sufficient quality.

Another important issue we found was to implement the induced sputum in our service. Although we followed straightly a standardized protocol^5^ almost 40% of the first study sample could not achieve adequate sputum to do the analyzes. This group’s characteristics had no statistical differences when compared to who expectorated adequate sputum. The proportion of inadequate samples in our research was higher than other studies^5,21,22^, probably because induced sputum to investigate inflammatory features is laborious as it demands time, trained professionals, and a qualified laboratory, able to process and count inflammatory cells^7^. It is not performed in our daily practice.

In conclusion, this cross-sectional study suggested that the finding of sputum eosinophilia in a population with severe asthma and in use of high doses of inhaled steroids was not related to asthma control, neither with health-related quality of life.

### Supplementary Information

Supplementary Information 1.

Supplementary Information 2.

## Data Availability

The datasets generated and/or analysed during the current study are available in the file “datasets.xlsx”, which is attached on “Supplementary Material”.
