# Supplementary material for: Asthma control and sputum eosinophils in adult patients: a cross-sectional study in southern Brazil
Source: Sci Rep. 2023 Dec 5;13:21464. doi: 10.1038/s41598-023-48381-1 (PMC10697938; doi:10.1038/s41598-023-48381-1)
Supplement: Supplementary file 2 — Supplementary Information 2. [file 41598_2023_48381_MOESM2_ESM.docx]

Supplement 1.

Induced sputum technique:

The study population was submitted to sputum induction to evaluate cellularity. The technique was adapted using Pizzichini et. Al methods(4).

Sputum induction was realized at Hospital de Clínicas de Porto Alegre Pulmonary Physiology Lab, analyzed at Cytopathology Service of the same hospital, by a graduated cytopathologist.

First, the technique starts with basal FEV1 measure, using spirometry. Second, the patient inhales 200mcg of salbutamol. After 20 minutes, FEV1 is measured one more time. If FEV1 is <70% of predicted, it can be inducted sputum using 7ml of saline 0,9% and RespiraMax ultrasonic nebulizer. If FEV1 is >= 70%, sputum is induced using 7ml of saline 3% and RespiraMax ultrasonic nebulizer. FEV1 is measured again. If it falls more than 20% of basal, the test is interrupted. If FEV1 measure falls between 10 and 20%, we replicate previous step of inhalation. If it falls last than 10%, the patient is instructed to mouthwash, drink water and blow-nose. After, the instruction is to cough and to deposit sputum on a clean pot.

This procedure is replicate with 4% and 5% saline.
